# Reactive Acrylamide-Modified DNA Traps for Accurate Cross-Linking with Cysteine Residues in DNA–Protein Complexes Using Mismatch Repair Protein MutS as a Model

**DOI:** 10.3390/molecules27082438

**Published:** 2022-04-10

**Authors:** Mayya V. Monakhova, Elena A. Kubareva, Kirill K. Kolesnikov, Viktor A. Anashkin, Egor M. Kosaretskiy, Maria I. Zvereva, Elena A. Romanova, Peter Friedhoff, Tatiana S. Oretskaya, Timofei S. Zatsepin

**Affiliations:** 1 A.N. Belozersky Institute of Physico-Chemical Biology, Lomonosov Moscow State University, Leninskye Gory 1, 119991 Moscow, Russia; kubareva@belozersky.msu.ru (E.A.K.); victor_anashkin@genebee.msu.ru (V.A.A.); romanova@belozersky.msu.ru (E.A.R.); oretskaya@belozersky.msu.ru (T.S.O.); 2 Department of Chemistry, Lomonosov Moscow State University, Leninskye Gory 1, 119991 Moscow, Russia; k.kolesnikov@krytex.pro (K.K.K.); zvereva@genebee.msu.ru (M.I.Z.); tsz@yandex.ru (T.S.Z.); 3 Department of Bioengineering and Bioinformatics, Lomonosov Moscow State University, Leninskye Gory 1, 119991 Moscow, Russia; megametagross@outlook.de; 4 Institute for Biochemistry, FB 08, Justus Liebig University, Heinrich-Buff-Ring 17, D-35392 Giessen, Germany; peter.friedhoff@chemie.bio.uni-giessen.de

**Keywords:** regioselectivity, DNA modification, DNA–protein complex, modified oligonucleotide, crosslinking, DNA mismatch repair, MutS

## Abstract

Covalent protein capture (cross-linking) by reactive DNA derivatives makes it possible to investigate structural features by fixing complexes at different stages of DNA–protein recognition. The most common cross-linking methods are based on reactive groups that interact with native or engineered cysteine residues. Nonetheless, high reactivity of most of such groups leads to preferential fixation of early-stage complexes or even non-selective cross-linking. We synthesised a set of DNA reagents carrying an acrylamide group attached to the C5 atom of a 2′-deoxyuridine moiety via various linkers and studied cross-linking with MutS as a model protein. MutS scans DNA for mismatches and damaged nucleobases and can form multiple non-specific complexes with DNA that may cause non-selective cross-linking. By varying the length of the linker between DNA and the acrylamide group and by changing the distance between the reactive nucleotide and a mismatch in the duplex, we showed that cross-linking occurs only if the distance between the acrylamide group and cysteine is optimal within the DNA–protein complex. Thus, acrylamide-modified DNA duplexes are excellent tools for studying DNA–protein interactions because of high selectivity of cysteine trapping.

## 1. Introduction

Understanding sequence-specific DNA–protein contacts at the molecular level is key to the development of synthetic molecules that can either block the formation of such contacts or affect DNA–protein recognition. For example, targeting a binding pocket of transcription factors has been proposed for cancer therapy [1]. A combined therapy involving several effectors of DNA recognition systems, such as transcription and DNA damage repair, may be even more beneficial [2]. Most of the structural data on DNA–protein complexes are obtained by X-ray analysis and cryo-electron microscopy (cryo-EM), but valuable information on the formation and functioning of DNA–protein complexes can be uncovered by affinity modification of DNA-binding proteins by means of oligonucleotides containing a reactive group(s) at a position determined beforehand [3,4,5,6,7,8]. Among the huge variety of reactive DNA derivatives, many studies have been focused on the modification of cysteine and lysine residues by selective chemical reactions or photoactivation of nitrobenzyl derivatives [9] or aryl azides or diazirines with broader reactivity [10].

Previously, we chose the well-characterised MutS protein, which belongs to the DNA mismatch repair (MMR) system [11], as a model binding partner to evaluate reactive DNAs. The MMR system corrects DNA damage by excising an extended single-stranded fragment of the newly synthesised DNA and then filling the resulting gap [12]. The key protein of this system—MutS—interacts with DNA at the first stage of MMR. This interaction with DNA is crucial for the regulation of MMR initiation [13,14]. MutS slides along DNA with the formation of multiple interim non-specific contacts with DNA until a mismatch or a damaged base is found. This event gives rise to the recognition complex and the sliding clamp state, which activates the next MMR steps [15]. These properties make the DNA–MutS complexes a perfect model for testing DNA reagents because these complexes involve different structures of both DNA and MutS. Several structures (based on X-ray data) of the recognition complex of MutS from *Escherichia coli* with DNA are available in the Protein Data Bank (PDB) at present [16,17,18,19,20,21]. Recently, four MutS–DNA complexes were characterised by cryo-EM [22]. On the other hand, less direct methods, such as Förster resonant energy transfer involving dually labelled probes or cross-linking approaches, can provide valuable information about different MutS–DNA complexes and their dynamic transitions [6,23].

Earlier, we introduced a 2′-pyridyl disulphide group into DNA to synthesise a reagent with a short linker (~7 Å) for affinity modification of the single-cysteine MutS variants MutS(A469C) and MutS(N497C) [21]. Pyridyl disulphides have several undoubted advantages. First, the group is very reactive towards thiols thereby leading to high yields of conjugates. In addition, the thiol–disulphide exchange reaction is reversible; therefore, one can release conjugate components in the presence of various reducing agents (e.g., dithiothreitol [DTT] or tris(2-carboxyethyl)phosphine [TCEP]). In the case of either MutS(A469C) or MutS(N497C), the conjugation with 2′-pyridyl disulphide DNA proceeds quantitatively in less than 5 min under mild conditions with an 80–90% yield. On the other hand, the high reactivity of such DNA reagents and non-specific binding to many proteins result in low selectivity. We observed a similar conjugation yield with an arbitrary DNA sequence; this means that this reagent is a poor candidate for specific cross-linking [21,23]. Therefore, the idea behind the present study was to test less reactive acrylamide derivatives of DNA to avoid nonspecific cross-linking.

Hocek et al. developed 2′-deoxycytidine-5′-triphosphates containing vinylsulphonamide and acrylamide residues for enzymatic incorporation into DNA that can be used for conjugation with proteins through the Michael addition [24]. Nonetheless, this excellent proof-of-concept study showed a rather low yield of the reaction of acrylamide-modified DNA with the p53 protein; accordingly, the main efforts were devoted to vinylsulphonamide derivatives there. Here, we synthesised and investigated alternative acrylamide derivatives of DNA for affinity modification of the single-cysteine variants MutS(A469C) and MutS(N497C) as model proteins: (i) modified oligonucleotides carrying an acrylamide group at the C5 position of uracil via a linker of various lengths (12–32 Å) and (ii) varying the distance between the modified base in double-stranded DNA and a G/T-mismatch allowed us to build DNA duplexes with optimal distances between a thiol group of cysteines and the acrylamide group. We showed for the first time the applicability of such DNA duplexes to regioselective cross-linking of a Cys residue (either Cys469 or Cys497) in the single-cysteine MutS variants. Good agreement of the results of the reaction in question with X-ray and Cryo-EM data gave us an opportunity to utilise the chemical method proposed in this work for precise testing of contacts in DNA–protein complexes.

## 2. Results and Discussion

### 2.1. The Design of Modified DNA Duplexes for Cross-Linking with the MutS Protein

**Figure 1:**
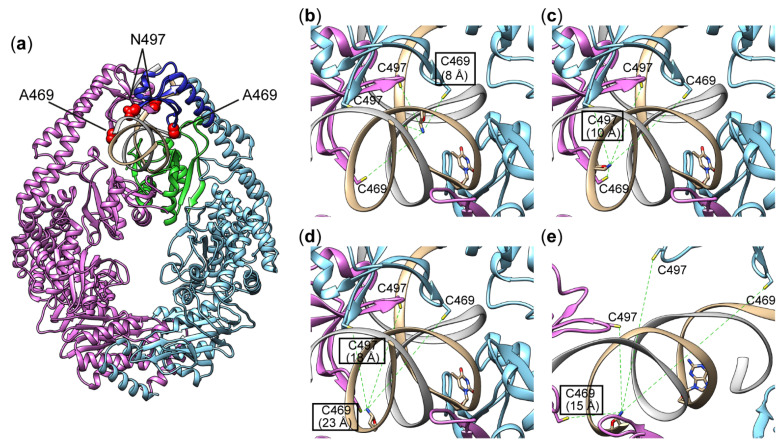
Complexes of MutS with DNA. An overview of the structure of MutS bound to DNA containing a mismatch (**a**). The clamp domain is highlighted in blue; the mismatch-binding domain is green, and A469 and N497 are red. The distances from the SG atom of C469 and C497 to the N atom of the heterocyclic base in the nucleotide located at the 5th (**b**), 8th (**c**) or 11th (**d**) position relative to the mismatch (see Figure 2). Panels (**b**–**d**) represent a detailed view of the structure of the MutS complex with a G/T-containing duplex, as determined by Cryo-EM (PDB ID 7AI6). Panel (**e**) is a fragment of a MutS complex with a canonical duplex (PDB ID 7AI5). The position of the modified base in the complex without the mismatch was determined by superposition of Cryo-EM structures with PDB IDs 7AI6 and 7AI5 (see Section 3.2 and Supplementary Materials). The two subunits of the protein are highlighted in blue (subunit A) and pink (subunit B), and the DNA is brown. The clamp domain is blue; the mismatch-binding domain is green, and A469 and N497 are red. Structures of native protein 7AI6 and 7AI5 have been described previously [22].

**Figure 2:**
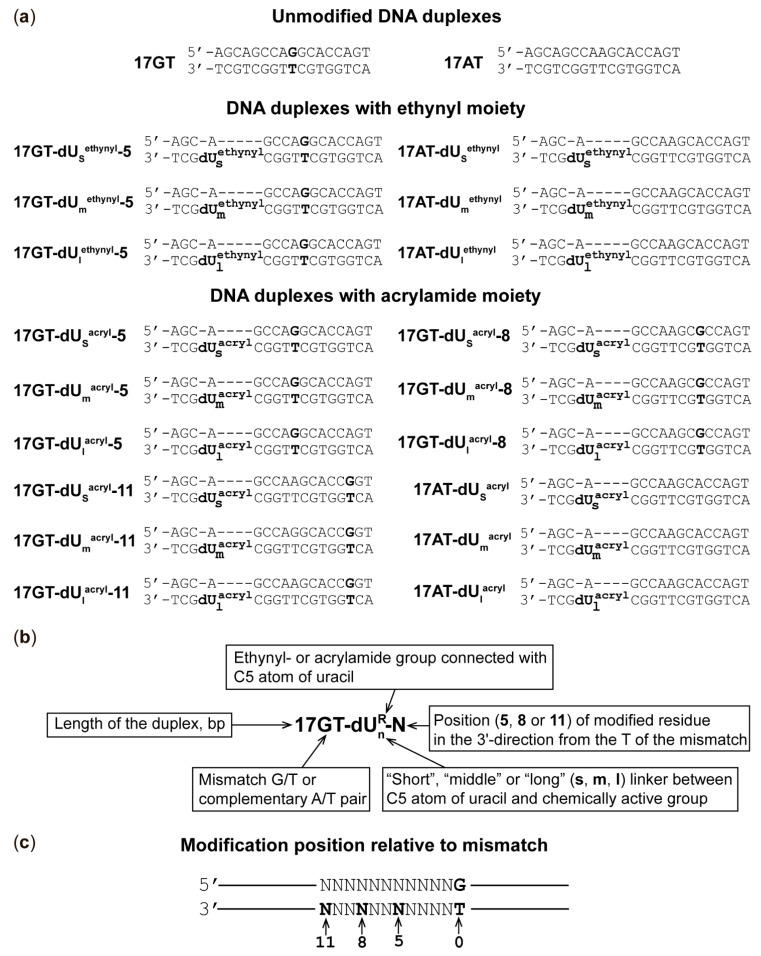
DNA duplexes used in this study. (**a**) The structure of DNA duplexes; modified dU residues are shown in Figure 3. (**b**) Explanation of the name of a duplex. (**c**) Illustration of positions of modified dU with respect to T of the G/T pair (position 0).

According to X-ray data, residues A469 and N497 are localised in the clamp domain in close proximity to the 5th and 8th nucleotides downstream from the thymidine of the mismatch (Figure 1a) [21,25]. To evaluate our cross-linking approach, previously created variants MutS(A469C) and MutS(N497C), each with a single cysteine residue in the clamp domain, were used [21,25]. Acrylamide-modified DNAs (Figure 2) with the same sequences as duplexes **17GT** and **17AT** (equivalent to **IV** and **V** in ref. [21]) were employed for cross-linking to compare the properties of acrylamide derivatives and 2′-pyridyl disulphide derivatives of DNA. We decided to introduce modified dU residues bearing the acrylamide group at various positions in the duplex strand containing T in a G/T mismatch (Figure 2a); this approach allowed us to direct the reactive groups into the major groove of the duplex.

**Figure 3:**
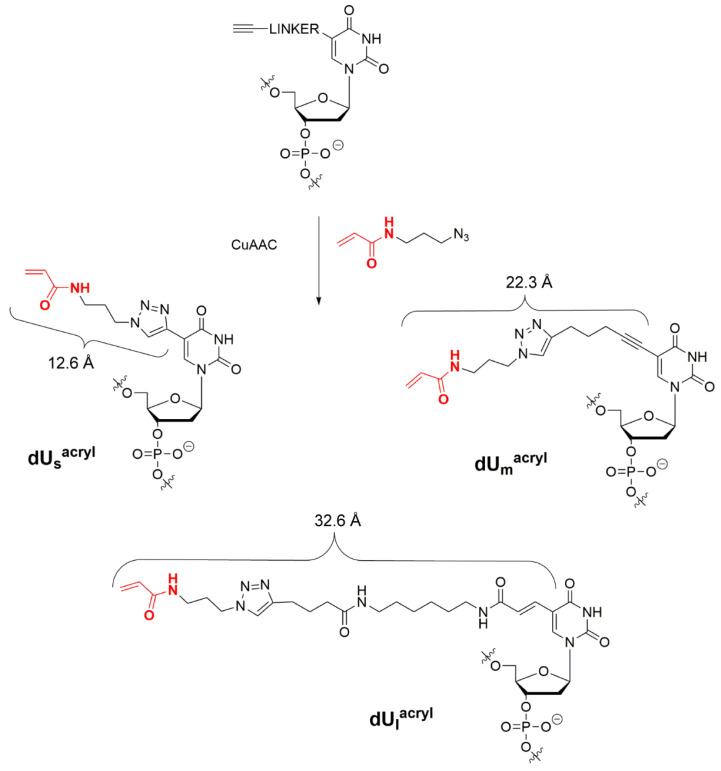
The scheme of the synthesis of oligonucleotides carrying the acrylamide group from ethynyl-containing oligonucleotides.

First, we measured distances between the Cys residue in the MutS variants and the C5 atom of thymidine at a selected position of DNA using published data obtained by X-ray crystallography and cryo-EM [16,17,18,19,20,21,22]. We chose the structure with PDB ID 3ZLJ as a starting point because it is based on the MutS–DNA complex with nucleotide cofactor ADP, which is necessary for efficient binding of MutS to a G/T mismatch and was also used in our research (Table S1). Nevertheless, because our sequence differs from the duplex used in the X-ray analysis, we modelled the corresponding DNA duplex in complex with MutS by means of the 3DNA software 2.0 web server (New York, NY, USA) [26]. Residues A469 and N497 were changed to Cys, and the distances between the sulphur atom of C469 or C497 and the C5 atom in thymine was calculated (5 and 8 nucleotides downstream of the T mismatch in the MutS–DNA complex; Table 1, Figure S1). On the basis of these data, we assumed that variation of the linker length between the C5 atom of uracil and the reactive acrylamide group from 12.6 (***s***) to 22.3 (***m***) and to 32.6 (***l***) Å will be enough for the direct contact of reactive groups and for the successful chemical reaction (see Section 3.2, Figure 3). 

Distance measurements in the modelled complex indicated (Table 1, Figure S1) that C469 of subunit A is the closest residue to the C5 uracil atom at the fifth position in the 3′ direction from the T mismatch in the MutS–DNA complex (Table 1). The cryo-EM structure of the 61-mer G/T-containing duplex with MutS in the presence of ADP did not allow us to identify individual heterocyclic bases owing to low resolution (6.9 Å). It was possible to estimate the positions of C469 and C497 with respect to the nitrogen atom of the modified nucleotide (Figure 1b). According to cryo-EM data, residues C469 and C497 interact with DNA on the minor-groove side, but the amino acid residue at position 469 is closer to DNA and more convenient for modification. On the basis of these data, we assumed that the modified nucleotide containing an acrylamide group connected via a linker of various lengths at this position of DNA duplexes **17GT-dU_s_^acryl^-5**, **17GT-dU_m_^acryl^-5** and **17GT-dU_l_^acryl^-5** should be close to the thiol group of cysteine and interact more efficiently with MutS(A469C).

A similar analysis of X-ray structures revealed that only C497 (from subunit A) is in close proximity to the C5 atom of thymidine at position 8 in the 3ʹ direction from the T of the mismatch (Table 1), and cryo-EM data confirmed this conclusion [22]. N497 in subunit A has a perfect position in the DNA major groove close to T(-8) (Figure 1c). Thus, DNA duplexes **17GT-dU_s_^acryl^-8**, **17GT-dU_m_^acryl^-8** and **17GT-dU_l_^acryl^-8** were constructed to test this assumption. We varied the position of the G/T mismatch to change its distance to the modified base pair without re-synthesising acrylamide-containing DNA (Figure 2).

Crystallographic data were obtained for the complexes of MutS with DNA duplexes in which the mismatch is flanked on the left by only eight base pairs (see Table 1 in [21]). In the cryo-EM structure, only 25 base pairs of the 61-bp DNA duplex are resolved [22]. According to our DNA–protein complex simulation data (see above) as well as cryo-EM data, amino acid residues at positions 469 and 497 are located at distances ~17 and ~22 Å from the nucleotide located at position 11 in the 3′ direction from the T mismatch (Figure 1d). Consequently, we decided to determine whether the DNA duplex with an acrylamide residue at this position can be cross-linked to either C469 or C497. For this purpose, duplexes **17GT-dU_s_^acryl^-11**, **17GT-dU_m_^acryl^-11** and **17GT-dU_l_^acryl^-11** were constructed in the same manner by placing the G/T mismatch further to the right end of the DNA (Table 1). It is known that MutS from *E. coli* has strong binding affinity for DNA termini [27]. Therefore, the recognition complex between MutS and DNA is not the only complex that can form in this case. On the other hand, if only the recognition complex can form, we hypothesised that the cross-linking of duplex **17GT-dU_m_^acryl^-11** or **17GT-dU_l_^acryl^-11** with MutS(N497C) should be the most efficient (Figure 1d). Position 497 is close to DNA on the side of the major groove, where the C5-acrylamide modification is located.

### 2.2. Synthesis of Oligonucleotides Containing a dU Residue Carrying an Acrylamide Group

One of the most common ways to label or conjugate proteins is the reaction of cysteine residues with α,β-unsaturated carbonyl compounds, mainly N-substituted maleimides (Michael reaction). Nevertheless, maleimides are incompatible with deprotection conditions of common phosphoramidite oligonucleotide synthesis; hence, they can be either additionally protected (which requires harsh deprotection [28]) or incorporated post-synthetically (which complicates construction of DNA reagents with short linkers). Less reactive acrylamide and methacrylamide moieties have been successfully incorporated into oligonucleotides during automated synthesis; this approach simplifies the production of such oligos for co-polymerisation within acrylamide for various applications [29,30], but acrylic derivatives are compatible only with ultra-mild deprotection conditions [29]. Hocek et al. have proposed using 2′-deoxycytidine triphosphate with an acrylamide group linked to the C5 atom for enzymatic DNA synthesis followed by cross-linking of the core domain of the p53 protein [24]. The yield of the conjugate was insufficient in that report; therefore, those authors switched their efforts to a more reactive vinylsulphone derivative. Moreover, the use of triphosphates for the synthesis of reactive DNA reagents imposes several limitations on the design of DNA ligands. For instance, the introduction of a single modified residue into a recognition site can be challenging for most of native sequences. 

Here we tried more robust post-synthetic derivatisation. First, we synthesised oligonucleotides using phosphoramidites of C5-modified 2′-deoxyuridines bearing either a terminal alkyne or amino group followed by acylation with hexynoic acid. After deprotection and initial purification, we conjugated *N*-(3-azidopropyl)acrylamide via the CuAAC reaction and thus prevented side reactions during the oligonucleotide synthesis and deprotection (Figure 3). As a result, we obtained a set of oligonucleotides with an acrylamide group attached to the C5 position of 2′-deoxyuridine via one of several linkers of various lengths: 5′-ACTGGTGCTTGGC**dU_s_^acryl^**GCT-3′ (***4***), 5′-ACTGGTGCTTGGC**dU_m_^acryl^**GCT-3′ (***5***) and 5′-ACTGGTGCTTGGC**dU_l_^acryl^**GCT-3′ (***6***). We estimated the length of linkers s, m and l at 12.6, 22.3 and 32.6 Å, respectively (Figure 3).

Oligonucleotides (***1***–***6***) (see Materials and Methods, Section 3.3) were next subjected to ^32^P labelling and annealing with templates to obtain either complementary or mismatch-containing DNA duplexes carrying either an ethynyl or acrylamide group (Figure 2). 

Thermal stability of the DNA duplexes (Table 2) was studied by means of changes in fluorescence of intercalating dye SYBR Green I as a function of temperature (Figure S2). The conformational transition of DNA from a double-stranded to a single-stranded state is accompanied by the release of the SYBR Green I molecule, thereby leading to a sharp decrease in the fluorescence intensity. Thermal stability of the complementary duplexes containing one of the three ethynyl modifications changed insignificantly, consistently with the literature data [31,32]. The introduction of a mismatch into a modified duplex decreased the melting temperature by 4–5 °C; the resultant melting temperature also slightly differed from that of the unmodified mismatch-containing duplex. Consequently, the introduced modifications at the C5 atom of uracil did not destabilise the DNA double helix.

### 2.3. MutS Binding to DNA Duplexes Carrying the Ethynyl Group

**Figure 4:**
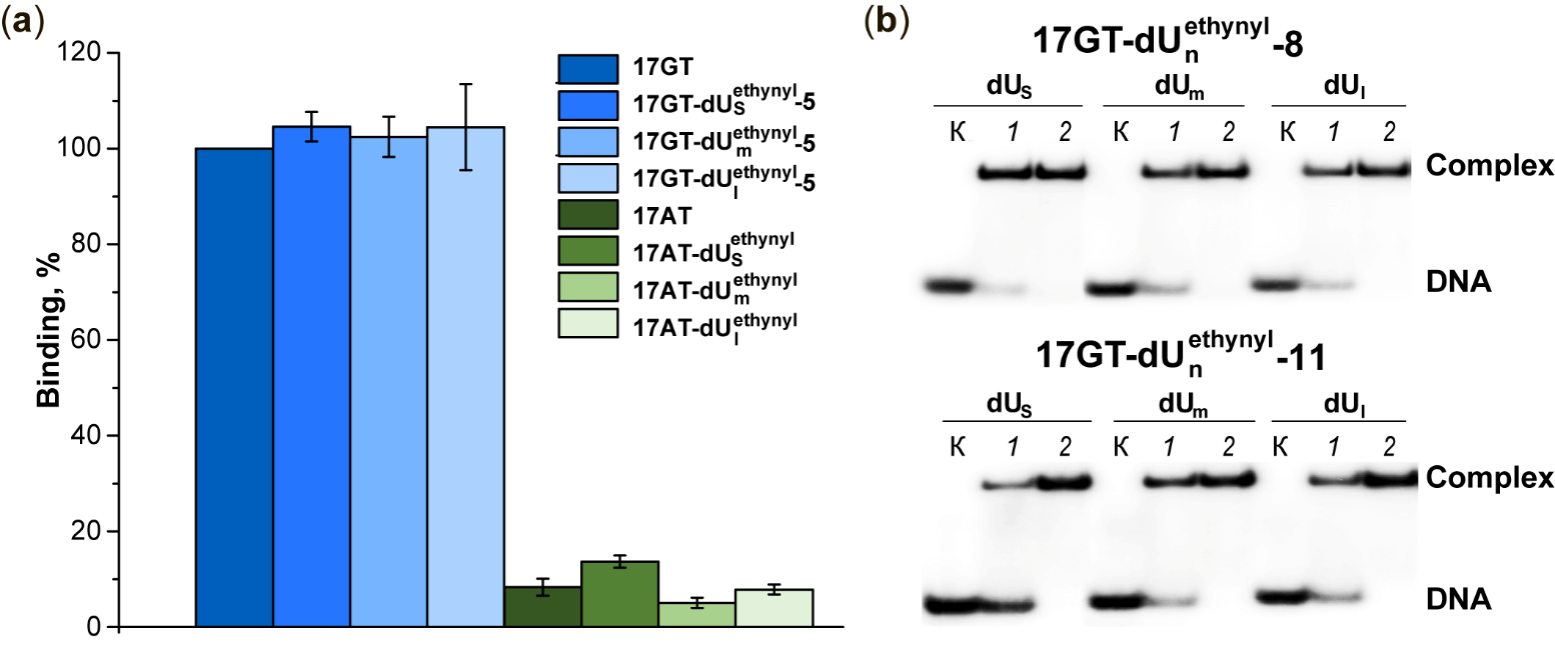
Binding of DNA duplexes to MutS(A469C). (**a**) Binding efficiency. Formation of the MutS(A469C) complex with an unmodified G/T-containing duplex is set to 100%. The standard deviation from the mean of at least three experiments is indicated; *p* < 0.05. (**b**) Analysis of complex formation between MutS(A469C) and ^32^P-labelled duplexes **17GT-dU_s_^ethynyl^-8**, **17GT-dU_m_^ethynyl^-8**, **17GT-dU_l_^ethynyl^-8** (top panel), **17GT-dU_s_^ethynyl^-11**, **17GT-dU_m_^ethynyl^-11** and **17GT-dU_l_^ethynyl^-11** (bottom panel) by an electrophoresis mobility shift assay (EMSA). DNA concentration was 0.5 µM. MutS(A469C) concentration was 1 µM (a, lanes *1*) or 2.5 µM (lanes *2*). Lanes K correspond to DNA without the protein. Autoradiograph of a 6% polyacrylamide gel.

In addition to mismatches, MutS can recognise some oxidative lesions and bulky adducts in DNA [33,34,35,36,37,38]. There was no detectable effect of the modified 2′-deoxyuridines on DNA duplex stability, but before cross-linking between a reactive DNA and the MutS protein, we checked whether the introduced modification can be recognised by MutS. For this purpose, we performed an electrophoretic mobility shift assay to compare the binding of MutS to a series of 17-bp DNA duplexes with a mismatch (G/T) or without (A/T) and with or without the indicated modifications (Figure 4).

A comparative analysis of the formation of a complex of MutS(A469C) with each of the five duplexes (**17AT-dU_s_^ethynyl^-5**, **17AT-dU_m_^ethynyl^-5**, **17AT-dU_l_^ethynyl^-5**, **17GT** and **17AT**) was carried out under the conditions of a five-fold excess of the protein (on the monomer basis) relative to DNA in order to attain maximal binding in ADP presence. Our results show that under these conditions, protein binding to ethynyl-containing DNA duplexes without the G/T pair does not exceed 10% relative to unmodified G/T-containing duplex **17GT** and is comparable to the complex formation between MutS(A469C) and canonical DNA **17AT** (Figure 4). We can conclude that the modified uridine residue is not recognised by MutS as damage and does not form a specific complex with it. Even though the introduced modification is in the major groove of DNA and MutS comes into contact with the mismatch from the minor groove [19,39], we tested whether the introduction of the modification affects the effective interaction of MutS with the mismatch. To this end, we compared the efficiency of complex formation between MutS(A469C) and a series of ethynyl-containing duplexes carrying the mismatch (**17GT-dU_s_^ethynyl^-5**, **17GT-dU_m_^ethynyl^-5** or **17GT-dU_l_^ethynyl^-5**) and with the mismatched duplex without the modification (**17GT**). There was no apparent difference between the modified and unmodified mismatch-containing DNA in the formation of the complex with MutS(A469C) (Figure 4). Because the introduction of any of the ethynyl modifications via a linker of various lengths into the DNA duplexes had no effect on MutS–DNA binding, the duplexes containing an acrylamide-modified DNA strand were suitable for cross-linking with the MutS protein.

It is known that the efficiency of DNA binding to the MutS protein depends on the number of bases flanking the mismatch [40]. In duplexes **17GT-dU_n_^ethynyl^-8** and **17GT-dU_n_^ethynyl^-11**, the mismatch is close enough to the ends of the DNA: 5 and 2 bp, respectively. We tested the binding of MutS(A469C) to such DNA with either a two-fold or five-fold excess of the protein. In the presence of 1 mM ADP and the five-fold excess of MutS, the formation of a complex between the protein and each modified duplex **17GT-dU_n_^ethynyl^-8** and **17GT-dU_n_^ethynyl^-11** was quantitative (Figure 4b). The efficiency of complex assembly under our conditions in both cases was comparable with the efficiency of MutS(A469C) binding to **17GT-dU_n_^ethynyl^-5**. Duplexes **17AT-dU_n_^ethynyl^-8** and **17AT-dU_n_^ethynyl^-11** served as a control for evaluating the capacity of the protein for non-specific binding and terminus binding. Their interactions with MutS(A469C) were significantly weaker in comparison with the corresponding modified DNA containing the mismatch. Therefore, MutS can give rise to specific complexes with **17GT-dU_n_^ethynyl^-8** and **17GT-dU_n_^ethynyl^-11**.

### 2.4. Interaction of MutS Variants with 17-mer DNA Duplexes Carrying the Acrylamide Group on a Linker of Various Lengths

**Scheme 1:**
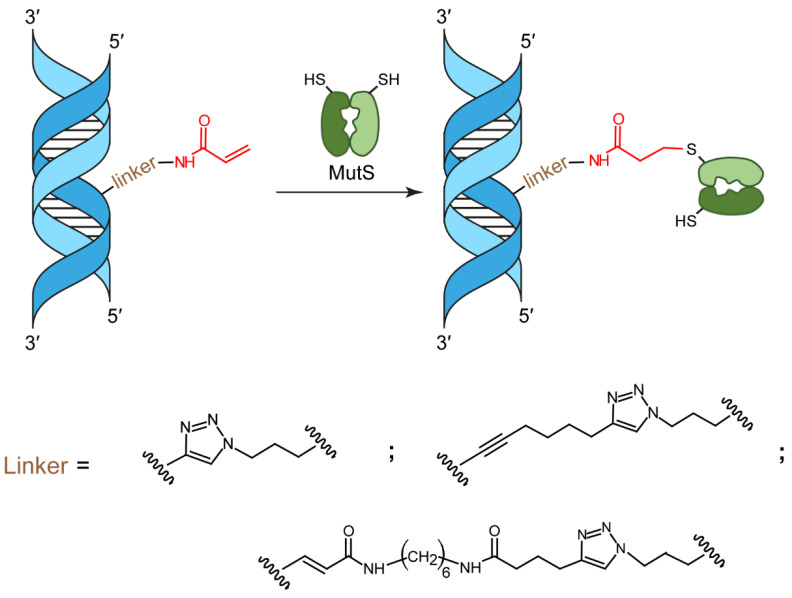
Affine modification of MutS with acrylamide containing DNA duplex.

**Figure 5:**
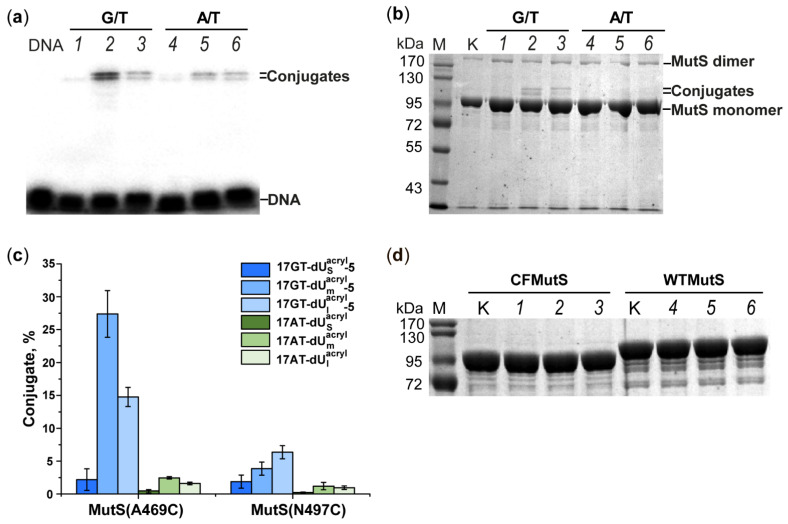
Cross-linking of MutS(A469C), MutS(N497C), CFMutS or WTMutS (5 μM on the monomer basis) with DNA duplex **17GT-dU_s_^acryl^-5**, **17GT-dU_m_^acryl^-5**, **17GT-dU_l_^acryl^-5**, **17AT-dU_s_^acryl^**, **17AT-dU_m_^acryl^** or **17AT-dU_l_^acryl^** (0.5 μM) containing a ^32^P label at the 5′ end of the modified strand. Analysis by 8% polyacrylamide gel electrophoresis (PAGE) with 0.1% of SDS. Autoradiograph (**a**) and photographs (**b**,**d**) of the gels stained with a Coomassie G250 solution. (**a**,**b**) Lanes *1*–*6*: products of MutS(A469C) cross-linking with duplex **17GT-dU_s_^acryl^-5**, **17GT-dU_m_^acryl^-5**, **17GT-dU_l_^acryl^-5**, **17AT-dU_s_^acryl^**, **17AT-dU_m_^acryl^** or **17AT-dU_l_^acryl^**, respectively. (**c**) Efficiency of a MutS(A469C) or MutS(N497C) reaction with **17GT-dU_n_^acryl^-5** or **17AT-dU_n_^acryl^**. The error bars represent the standard deviation of three independent experiments; *p* < 0.05. (**d**) Interaction of CFMutS (lanes *1*–*3*) or WTMutS (lanes *4*–*6*) with **17GT-dU_s_^acryl^-5**, **17GT-dU_m_^acryl^-5** or **17GT-dU_l_^acryl^-5**. Lanes K correspond to the protein without DNA; DNA lane: **17GT**, M: markers of protein molecular mass, kDa.

**Figure 6:**
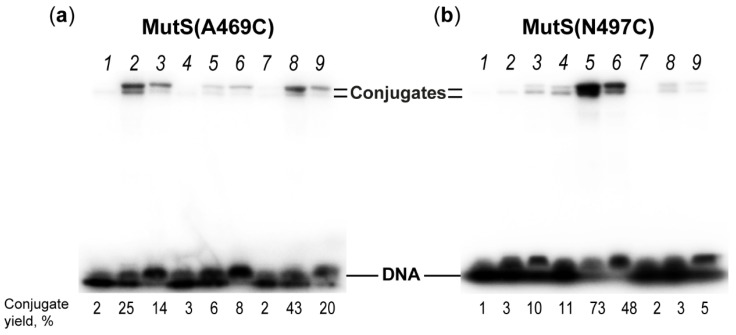
MutS(N469C) (**a**) or MutS(N497C) (**b**) cross-linking with **17GT-dU_s_^acryl^-5**, **17GT-dU_m_^acryl^-5** or **17GT-dU_l_^acryl^-5** (lanes *1*–*3*); **17GT-dU_s_^acryl^-8**, **17GT-dU_m_^acryl^-8** or **17GT-dU_l_^acryl^-8** (lanes *4*–*6*); or **17GT-dU_s_^acryl^-11**, **17GT-dU_m_^acryl^-11** or **17GT-dU_l_^acryl^-11** (lanes *7*–*9*). Conjugate yields (%) are indicated under the gel lanes. Protein concentration on the monomer basis was 5 µM, DNA: 0.5 µM. Duplexes contain a ^32^P label at the 5′ end of the modified strand. Autoradiograph of SDS-PAGE in an 8% gel.

Just like maleimides, acrylamides react with thiols of a protein through the Michael addition. Of the two single-cysteine MutS variants in our work, only MutS(A469C) reacted with a high yield with the duplexes containing an acrylamide-modified DNA strand (Scheme 1). Covalent capture of MutS(A469C) by 5′-^32^P-labelled DNA duplexes **17GT-dU_s_^acryl^-5, 17GT-dU_m_^acryl^-5** and **17GT-dU_l_^acryl^-5** (Table 1) was implemented at 37 °C in a buffer containing 1 mM ADP, which stabilises an initial DNA–protein complex [41]. To achieve a high yield of the conjugate, a 10-fold excess of the protein (on the monomer basis) relative to DNA was used (Figure 5 and Figure 6). By autoradiography and Coomassie G250 staining, we detected two additional bands with lower mobility in comparison to free DNA and MutS(A469C) (Figure 5a). They can be considered the products of cross-linking, and most likely the double band was due to high stability of DNA duplexes (T_m_ ≈ 80 °C): to be precise, the two bands may correspond to conjugates of MutS(A469C) with single-stranded DNA and with a DNA duplex (Figure 5a and Figure 6). Because initially, after SDS-PAGE in an 8% gel, we observed the emergence of the MutS dimer (>170 kDa) (Figure 5b), we added 1 mM TCEP to the reaction mixture to avoid concurrent MutS dimerisation during the cross-linking. Thiol-free TCEP reduces disulphide bonds as effectively as DTT does, but unlike DTT and other thiol-containing reducing agents, TCEP does not have to be removed before thiol-selective conjugation [6,7,8,21,23,42].

A quantitative analysis of the conjugation reaction indicated that MutS(A469C) reacted most efficiently with DNA containing the acrylamide group on the 22.3 Å linker (**17GT-dU_m_^acryl^-5**), as we expected according to the modelling data (Figure 5c). The yield of the conjugate of MutS(A469C) with the **17GT-dU_m_^acryl^-5** DNA duplex was more than 27% (Figure 5a, lane *2*). Obviously, the DNA reagent with linker ***m*** offers an optimal distance to Cys469, which is 10–13 Å according to our calculations (Table 1).

The efficiency of the conjugation of MutS(A469C) with any of the acrylamide-containing DNAs carrying the mismatch (**17GT-dU_s_^acryl^-5**, **17GT-dU_m_^acryl^-5** and **17GT-dU_l_^acryl^-5**) was significantly lower compared to that observed with a pyridyl disulphide-modified DNA duplex carrying the 2′-pyridyldithio group on dU at the same position (the maximum yield of conjugates was 27% in 2 h vs. a quantitative yield in 30 min). It is likely that due to the lower reactivity of acrylamide than that of the pyridyldithio group, the conjugation takes place only after the emergence of the DNA–protein recognition (specific) complex. Given that MutS(A469C) cross-linking to the duplex without the mismatch-**17AT-dU_m_^acryl^-**proceeded significantly less efficiently (<3%; Figure 5c) as compared to a similar DNA duplex carrying the 2′-pyridyldithio group (60% per protein), we believe that acrylamide derivatives of DNA have higher selectivity of conjugation. 

To assess the selectivity of the acrylamide-modified-DNA reagents towards the Cys residues close to DNA, the interaction of duplexes **17GT-dU_s_^acryl^-5**, **17GT-dU_m_^acryl^-5** and **17GT-dU_l_^acryl^-5** with MutS(N497C), CFMutS or wild-type MutS (WTMutS) was researched next. CFMutS, which does not contain Cys residues, did not produce a conjugate with any reactive DNA (Figure 5d). The same result was obtained with WTMutS, which contains six Cys residues per monomer. MutS(N497C) also manifested a low yield of conjugation with modified DNAs (Table 1, Figure 6b). It should be noted that duplex **17GT-dU_m_^acryl^-5** is optimal for MutS(N469C) cross-linking, even though distances from the C5 atom of dU modified at the 5th position to the SG atom of Cys469 and Cys467 are similar (10 vs. 14 Å, respectively), and linker ***m*** (22.3 Å) is two-fold longer. One can theorise that the accessibility of the acrylamide group (linker ***m***) in the DNA major groove is higher for Cys469 than for Cys497. At the same time, accessibility of the acrylamide group on the ‘short’ linker (***s***) is obviously not sufficient. Consequently, the acrylamide-containing DNA ligands helped us to demonstrate that as compared to position 497, position 469 is closer to the 5th nucleotide in the 3′ direction from the T residue of the mismatch. This result is consistent with the structural data on the DNA complex with MutS in the recognition state.

The next step for confirming the selectivity of the reaction was covalent binding of either MutS(N497C) or MutS(A469C) to duplex **17GT-dU_s_^acryl^-8, 17GT-dU_m_^acryl^-8** or **17GT-dU_l_^acryl^-8**. In these duplexes, the modification was placed at position 8 in the 3′ direction from the T of the G/T mismatch (Figure 2). According to the structure of the initial MutS–DNA recognition complex, the nucleotide at this position is close only to the cysteine residue of MutS(N497C) (Table 1, Figure 1c). We found that this MutS variant effectively produces conjugates with **17GT-dU_s_^acryl^-8**, **17GT-dU_m_^acryl^-8** and **17GT-dU_l_^acryl^-8** (Figure 6). Moreover, the yield of the reaction product in the case of DNA with the acrylamide group on the medium linker was very high: it reached 76%, which is comparable to the conjugate yield after the interaction of MutS(N497C) with DNA containing dU carrying the 2′-pyridyl disulphide group at the same position. MutS(A469C) was almost unreactive with duplexes **17GT-dU_s_^acryl^-8**, **17GT-dU_m_^acryl^-8** and **17GT-dU_l_^acryl^-8**, apparently owing to the suboptimal arrangement of Cys and the modification in DNA (Table 1, Figure 1c). Cys469 is located in the minor DNA groove and is not accessible to the acrylamide group even with long linker ***m*** or ***l*** because of the steric hindrance within the DNA–protein complex. 

**Figure 7:**
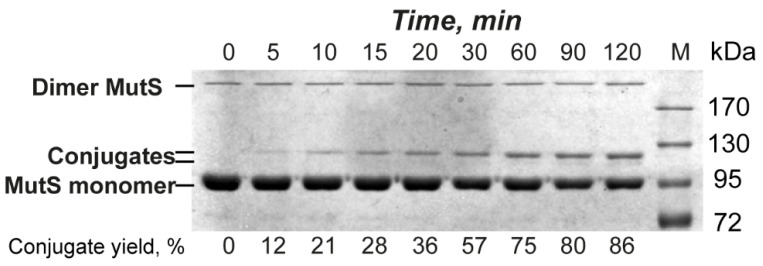
Analysis of MutS(N497C) (5 μM on the monomer basis) cross-linking with the **17GT-dU_m_^acryl^-8** DNA duplex (0.5 μM) by SDS-PAGE in an 8% gel. Reaction conditions: 0–120 min, 37 °C. Conjugate yields (%) are indicated under the gel lanes. The gel was stained with a Coomassie G250 solution. M: markers of protein molecular mass, kDa.

Given that we managed to achieve a high yield of the conjugate in the reaction of MutS(N497C) with **17GT-dU_m_^acryl^-8**, we decided to investigate the kinetics and showed that the conjugate synthesis proceeds rather slowly. The highest yield of the conjugate of MutS(N497C) with **17GT-dU_m_^acryl^-8** was obtained in 2 h (Figure 7), while longer reaction times led to inactivation of MutS and its variants.

The interaction of the MutS variants with position 11 in DNA in the 3′ direction from the T mismatch was intriguing for two reasons: (1) the absence of X-ray data characterising this interaction and (2) the proximity of the mismatch to the end of DNA and therefore competition between two processes: binding of MutS to a non-complementary base pair and binding to the DNA right-hand end (Figure 2). Accordingly, three modified DNA duplexes were synthesised containing dU carrying the acrylamide group at this position on a linker of various lengths: **17GT-dU_s_^acryl^-11**, **17GT-dU_m_^acryl^-11** or **17GT-dU_l_^acryl^-11** (Figure 2). As suggested above, the cross-linking of **17GT-dU_m_^acryl^-11** or **17GT-dU_l_^acryl^-11** with MutS(N497C) may be the most efficient. Our findings about the interaction of these DNAs with variants MutS(N497C) and MutS(A469C) are presented in Figure 6 and Table 1. 

Notably, only MutS(A469C) reacted with such DNAs. It should be pointed out that in the case of modified DNA with linker ***m*** or ***l*** (22–33 Å in length), a 20–40% yield was observed. For DNA with the acrylamide group on the ‘short’ linker, the reaction product was almost absent, indicating that Cys residues were inaccessible to the acrylamide group. This finding contradicts the structure of MutS bound to DNA carrying a single mismatch as revealed by cryo-EM (PDB ID 7AI6, Figure 1d) [22]. We have previously shown that a thiol-containing reactive group at the 3′ end of the duplex traps both variants MutS(N497C) and MutS(A469C) regardless of the mismatch presence at the 8th position from the modified terminus [25]. Nevertheless, these data did not explain our results either. Accordingly, we analysed the cryo-EM structure of MutS bound to perfectly matched DNA (PDB ID 7AI5, Figure 1e). This structure was superposed on the structure of the complex of MutS with G/T-containing DNA (PDB ID 7AI6). This manoeuvre enabled us to identify the location of the modified dU in structure 7AI5. In such a non-specific complex, position 469 in MutS is located at ~15 Å from the reactive nucleoside, while 497 is far away. Nonetheless, the yields of MutS(A469C) cross-linking to reactive duplexes **17AT-dU_s_^acryl^, 17AT-dU_m_^acryl^** and **17AT-dU_l_^acryl^** (without the mismatch) were low (Figure 5). Thus, our results could not be explained only by the non-specific binding that was observed for the interaction of MutS(A469C) with **17GT-dU_m_^acryl^-11** or **17GT-dU_l_^acryl^-11**. Moreover, the yield of MutS(A469C) cross-linking to DNA with the modification at the 11th position on linker ***m*** (**17GT-dU_m_^acryl^-11**) was even higher (42%) as compared with the yield of MutS(A469C) cross-linking (27%) to **17GT-dU_m_^acryl^-5**, where the G/T pair is far from the terminus. Therefore, the MutS–DNA recognition complex should form; this was confirmed by the effective non-covalent binding of MutS(A469C) to **17GT-dU_n_^ethynyl^-11** (see Section 2.3). Perhaps in this case, the MutS recognition complex cannot form completely due to the proximity of the mismatch to the ends of the DNA, and therefore MutS may interact with such DNA in a special not yet identified manner. 

**Figure 8:**
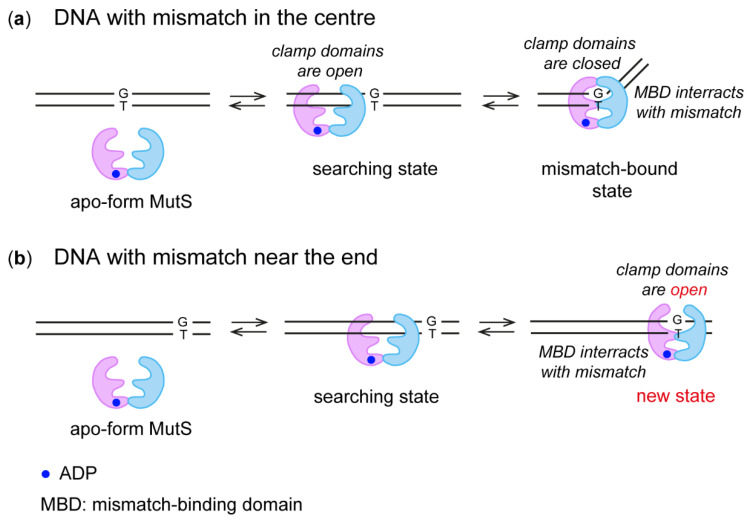
MutS binding to DNA with a mismatch in the middle (**a**) or near a terminus of the duplex (**b**).

In the recognition complex with DNA, the MutS mismatch-binding domain (MDB) interacts with the G/T pair, while the clamp domain is in a closed conformation (Figure 8a), as described in detail before [16,17,18,19,20,21]. In the scanning complex—when MutS is searching for a mismatch in DNA—the clamp domain is in an open state [22]. Such a conformation is also common for the MutS apo-form [39,43]. We suppose that during the MutS(A469C) interaction with **17GT-dU_m_^acryl^-11**, the complex was fixed when MutS MDB was bound to the G/T pair as in the recognition complex, but the MutS clamp domain had a movable open conformation as in the scanning complex (Figure 8b). The movability of the clamp domain has been demonstrated in some studies by X-ray crystallography [20,39,44] and recently by cryo-EM [22,45]. It is possible that such a MutS state does not permit a kink in DNA. Consequently, according to our cross-linking data, we propose the existence of a previously undescribed MutS state in the complex with DNA.

## 3. Materials and Methods

All reagents for oligonucleotide synthesis and purification and protein experiments were of the highest grade and used as received without further purification. Phosphoramidites 5-ethynyl-5′-O-(4,4′-dimethoxytrityl)-2′-deoxyuridine (dU_s_^ethynyl^), 5-(octane-1,7-diinyl)-5′-O-(4,4′-dimethoxytrityl)-2′-deoxyuridine (dU_m_^ethynyl^), 5-(2-[6-trifluoroacetamidohexyl]-3-acroylamido)-5′-O-(4,4′-dimethoxytrityl)-2′-deoxyuridine (dU_l_^ethynyl^), hexynoic acid NHS ester and *N*-(3-azidopropyl)acrylamide were purchased from Lumiprobe (LLC, Moscow, Russia).

### 3.1. Proteins

MutS and its variants were expressed and purified as described previously [46] and were stored in buffer A (10 mM HEPES-KOH pH 7.9, 200 mM KCl, 1 mM EDTA and 10% [*v*/*v*] of glycerol) at −80 °C. 

### 3.2. Modelling of Modified DNA and DNA–Protein Complexes

ChemSketch (ACD Labs; Toronto, ON, Canada) was employed to construct a 3D model of modified dU carrying the acrylamide group on a linker of various lengths. UCSF Chimera 1.13.1 software was used to estimate the linkers’ length between the uracil C5 atom and the C atom of the CH2 group in the acrylamide moiety and to compute the distance between modified dU (C5 position) in DNA and C469/C497 in MutS (SG atom). A model of the MutS–DNA complex with the G/T pair at a distance of 8 or 11 bp from the modified base was constructed on the WEB 3DNA 2.0 web server (New York, NY, USA) (http://web.x3dna.org (accessed on 2 July 2019)) [26] by means of the ‘composite’ function (structure with PDB ID 3ZLJ served as a template). With the help of the ‘mutated’ function, DNA sequence in the model was corrected to match experimental DNAs (for more details, see Supplementary Materials). 

### 3.3. The Synthesis of Oligonucleotides

This was carried out on an ABI 3400 DNA synthesiser (Applied Biosystems, Waltham, MA, USA) by the standard phosphoramidite method, with minor modifications for the synthons carrying the ethynyl or acrylamide group: the coupling time was increased up to 15 min. The oligonucleotides were deprotected using AMA (ammonium hydroxide/40% aqueous methylamine at 1:1, *v*/*v*) for 30 min at 65 °C, analysed by reversed-phase high-performance liquid chromatography (HPLC) and purified by polyacrylamide gel electrophoresis (PAGE) in a denaturing (7 M urea) gel, followed by isolation from the gel by means of the Elutrap instrument (Whatman, Marlborough, MA, USA). Purification by HPLC was conducted on an ÄKTA Purifier (GE Healthcare, Chicago, IL, USA) equipped with a Jupiter C18 column (Phenomenex, California, USA, Jupiter 5 μm, 300 Å, 250 × 4.6 mm) and a UV-Vis detector. To obtain oligonucleotide **17GT-dU_l_^ethynyl^**, we performed acylation of the amino precursor with hexynoic acid NHS ester (10 equivalents) in 200 mM carbonate buffer (pH 8.5, 50% of DMSO) overnight. Acrylamide derivatives were synthesised by CuAAC of ethynyl oligonucleotides with *N*-(3-azidopropyl)acrylamide via a previously devised procedure [47]. Oligonucleotides were characterised by liquid chromatography coupled with mass spectrometry on Ultimate 3000-LCQ instrument (Thermo Fisher Scientific, Waltham, MA, USA) as described previously [48]: 5′-ACTGGTGCTTGGC**dU_s_^ethynyl^**GCT-3′ (***1***, M_calcd_/M_found_ 5218.4/5223.2), 5′-ACTGGTGCTTGGC**dU_m_^ethynyl^**GCT-3′ (***2****,* M_calcd_/M_found_ 5299.5/5304.1), 5′-ACTGGTGCT-TGGC**dU_l_^ethynyl^**GCT-3′ (***3***, M_calcd_/M_found_ 5456.7/5463.0), 5′-ACTGGTGCTTGGC**dU_s_^acryl^**GCT-3′ (***4***, M_calcd_/M_found_ 5372.6/5375.4), 5′-ACTGGTGCTTGGC**dU_m_^acryl^**GCT-3′ (***5****,* M_calcd_/M_found_ 5453.7/5457.0) and 5′-ACTGGTGCT-TGGC**dU_l_^acryl^**GCT-3′ (***6***, M_calcd_/M_found_ 5610.9/5613.2).

### 3.4. The Effect of Temperature on the Stability of DNA Duplexes Carrying the Ethynyl Group 

Thermal stability of the modified DNAs was estimated on RT-PCR machine ANK-16/32 (The Institute for Analytical Instrumentation Russian Academy of Science, Novosibirsk, Russia) by monitoring fluorescence changes of the SYBR Green I dye at 520 nm. SYBR Green I is a sensitive fluorescent indicator of double-stranded DNA. The excitation wavelength of the SYBR Green I complex with DNA is 488 nm (blue light), and the emission wavelength is 522 nm (green light). The melting curves reflect the temperature dependence of the fluorescence intensity (I_fl_) of the complex of DNA with SYBR Green I. 

The temperature programme was as follows: incubation at 30 °C for 15 min and heating from 30 to 95 °C for 130 min. The measurement was carried out in buffer B (20 mM HEPES-KOH pH 8.0, 125 mM KCl, 5 mM MgCl_2_, 0.05% of glycerol, 0.01% of Tween 20, less than 0.5 μM SYBR Green I [dilution 1:10,000, *v*/*v*]). The concentration of DNA duplexes was 2 μM, and the volume of each sample was 40 μL. The obtained melting curves in integral form were processed in the Origin 8.1 software, and the curves were fitted to the Boltzmann sigmoid function. From the obtained integral ‘melting’ curve, the ‘melting’ temperature (T_m_) was determined as the temperature at which the drop in the SYBR Green I fluorescence intensity was equal to half the maximum. The integral curve of the dependence of the fluorescence intensity of the DNA duplex on temperature was also converted into a differential form, which helped to determine T_m_ more accurately, as the temperature at the maximum of the differential curve. The standard deviation was calculated from the data of at least four independent experiments.

### 3.5. ^32^P Labelling of the Oligonucleotides and Preparation of DNA Duplexes 

^32^P was introduced into oligonucleotides using T4 polynucleotide kinase (10 U) and [γ-^32^P]ATP (200 nM) in 10 μL of a buffer (50 mM Tris-HCl pH 7.6, 10 mM MgCl_2_, 5 mM DTT and 100 μM spermidine) for 30 min at 37 °C. Unreacted [γ-^32^P]ATP was removed by gel filtration on an Ultra MicroSpin G-50 column (GE Healthcare, Chicago, IL, USA). ^32^P-labelled DNA duplexes were prepared by successive denaturation/renaturation of the complementary strands in equimolar amounts in H_2_O and stored at −20 °C. 

### 3.6. Complex Formation between the MutS(A469C) Variant and Ethynyl-Containing DNAs 

A mixture of a ^32^P-labelled DNA duplex (0.5 μM) with MutS(A469C) (1 or 2.5 μM on the monomer basis) was incubated at 37 °C for 20 min in buffer C (20 mM HEPES-KOH pH 8.0, 125 mM KCl, 1 mM ADP and 5 mM MgCl_2_). The samples were analysed by electrophoresis in a 6% polyacrylamide gel in TAE buffer (40 mM Tris-CH_3_COOH pH 7.5, 1 mM EDTA) for 3 h at 4 °C (electrophoresis mobility shift assay). Visualisation of bands on the gel containing the ^32^P label and acquisition of the data were performed using FLA-3000 (Fujifilm, Japan) and Typhoon FLA 9500 (GE Healthcare, Chicago, IL, USA) followed by data analysis in the TotalLab TL120 2.01 software (GE Healthcare, Chicago, IL, USA). Each measurement was performed at least three times.

### 3.7. Cross-Linking of MutS Protein Variants with Reactive DNA Containing the Acrylamide Group 

A variant of MutS (5 μM on the monomer basis) was incubated with a labelled DNA duplex carrying the acrylamide group (0.5 μM) at 37 °C for 2 h in buffer C supplemented with 1 mM TCEP (to decrease protein dimerisation via a disulphide bond). Reaction products were separated by 8% PAGE with 0.1% of SDS. The gel contained a 4% concentrating layer. The gels after drying were scanned on the FLA-3000 device in a BAS Cassette2 2340 with a screen sensitive to β-radiation (Fujifilm, Japan). The cross-linking yield was estimated as the ratio of intensity of a ^32^P-labelled DNA band of the conjugate to the total intensity of the bands containing the ^32^P-labelled DNA in a gel lane. Each measurement was performed at least three times; standard error (SE) did not exceed 8–10%. Standard deviation from at least three identical experiments was computed in Origin (OriginLab, Northampton, MA, USA, https://www.originlab.com/, accessed on 21 September 2021). After that, the gel was placed in water and stained with a Coomassie Brilliant Blue G250 solution to visualise the protein. 

## 4. Conclusions

We created a set of new DNA reagents carrying an acrylamide group attached at the C5 position of dU via various linkers. According to the known structure of the MutS–DNA initial recognition complex, we constructed a series of DNA duplexes with the acrylamide group directed into the major groove at several positions. The optimal reactivity of the acrylamide group and variation of linker length and duplex arrangement allowed us to demonstrate selectivity of conjugation via residues Cys469 and Cys497 located in the vicinity of DNA. Even the use of a relatively long linker (total length ~22.3 Å) at an optimal position gave a regioselective reaction with MutS(N497C) with a >70% yield. On the basis of our cross-linking results, we can hypothesise that a previously undescribed ‘hybrid’ MutS state is present in the complex with DNA. The excellent agreement of our cross-linking findings with X-ray and cryo-EM data makes the newly developed reagents promising for research on structural features of DNA–protein complexes.

## Figures and Tables

**Table 1:** Calculated distances from the SG atom of Cys469 or Cys497 in MutS to C5 of dU at some positions in DNA and the yield of MutS–DNA conjugates.

Position of Cys Residue in MutS		Distance between Indicated Cys and DNA, Å	Conjugate MutS–DNA Yield, % Linker (Its Length, Å)		
			s (12.6 Å)	m (22.3 Å)	l (32.6 Å)
*Modification at 5th position from mismatch*					
**469**	Subunit A	10	2 ± 1	27 ± 4	15 ± 1
	Subunit B	13			
**497**	Subunit A	14	2 ± 1	4 ± 1	6 ± 1
	Subunit B	20			
*Modification at 8th position from mismatch*					
**469**	Subunit A	19	1	2 ± 1	5 ± 1
	Subunit B	12			
**497**	Subunit A	9	12 ± 1	76 ± 2	53 ± 6
	Subunit B	25			
*Modification at 11th position from mismatch*					
**469**	Subunit A	31	2 ± 1	42 ± 2	17 ± 3
	Subunit B	19			
**497**	Subunit A	19	2 ± 1	4 ± 2	3 ± 1
	Subunit B	37			

**Table 2:** Thermal stability of modified DNA duplexes *.

Duplex	T_m_, °C
**17AT**	81.6 ± 0.3
**17AT-dU_s_^ethynyl^**	80.7 ± 0.3
**17AT-dU_m_^ethynyl^**	81.3 ± 0.3
**17AT-dU_l_^ethynyl^**	80.6 ± 0.5
**17GT**	77.1 ± 0.3
**17GT-dU_s_^ethynyl^-5**	77.5 ± 0.1
**17GT-dU_m_^ethynyl^-5**	76.9 ± 0.3
**17GT-dU_l_^ethynyl^-5**	76.6 ± 0.1

* Melting temperature (T_m_) and standard deviation were calculated from four independent experiments.

## Data Availability

Not applicable.

## Supplementary Materials

The following supporting information can be downloaded at: https://www.mdpi.com/article/10.3390/molecules27082438/s1, Table S1: Calculated distances (Å) from amino acid residues of MutS to the C5 atom of thymine at 5th and 8th positions from T of the mismatch in X-ray structure; Figure S1: The dependence of fluorescence intensity of the DNA complex with SYBR Green on the temperature, Figure S2: A model of the MutS–DNA complex based on crystal structure of the complex.
