# Supplementary material for: Reactive Acrylamide-Modified DNA Traps for Accurate Cross-Linking with Cysteine Residues in DNA–Protein Complexes Using Mismatch Repair Protein MutS as a Model
Source: Molecules. 2022 Apr 10;27(8):2438. doi: 10.3390/molecules27082438 (PMC9031232; doi:10.3390/molecules27082438)
Supplement: Supplementary file 1 [file molecules-27-02438-s001.zip › molecules-1653720-supplementary.pdf]

SUPPLEMENTARY INFORMATION

Analysis of MutS-DNA interactions

The models of MutS-DNA complex were obtained based on 3ZLJ structure [20]. There is the A residue in the 5th position from G/T mismatch in 3'-direction in original structure. In order to calculate the distance, we flipped this A-T pair. To evaluate the distances from modification in 8th and 11th positions G/T mismatch in 3ZLJ structure was shifted in 3'-direction by replacement of the A residue to G in "top" strand for both cases (the modification is in the same position). In the case of modification in 8th or 11th position 2 or 3 random nucleotides were added in 3'-direction from the G/T pair to keep all MutS-DNA contacts for correct model building. The original G/T pair was changed to G/C pair. All procedures were made using web server WEB 3DNA 2.0. After construction of the complexes residues A469 and N497 were changed to Cys in Chimera 1.13.1 program followed by energy optimization.

(a)

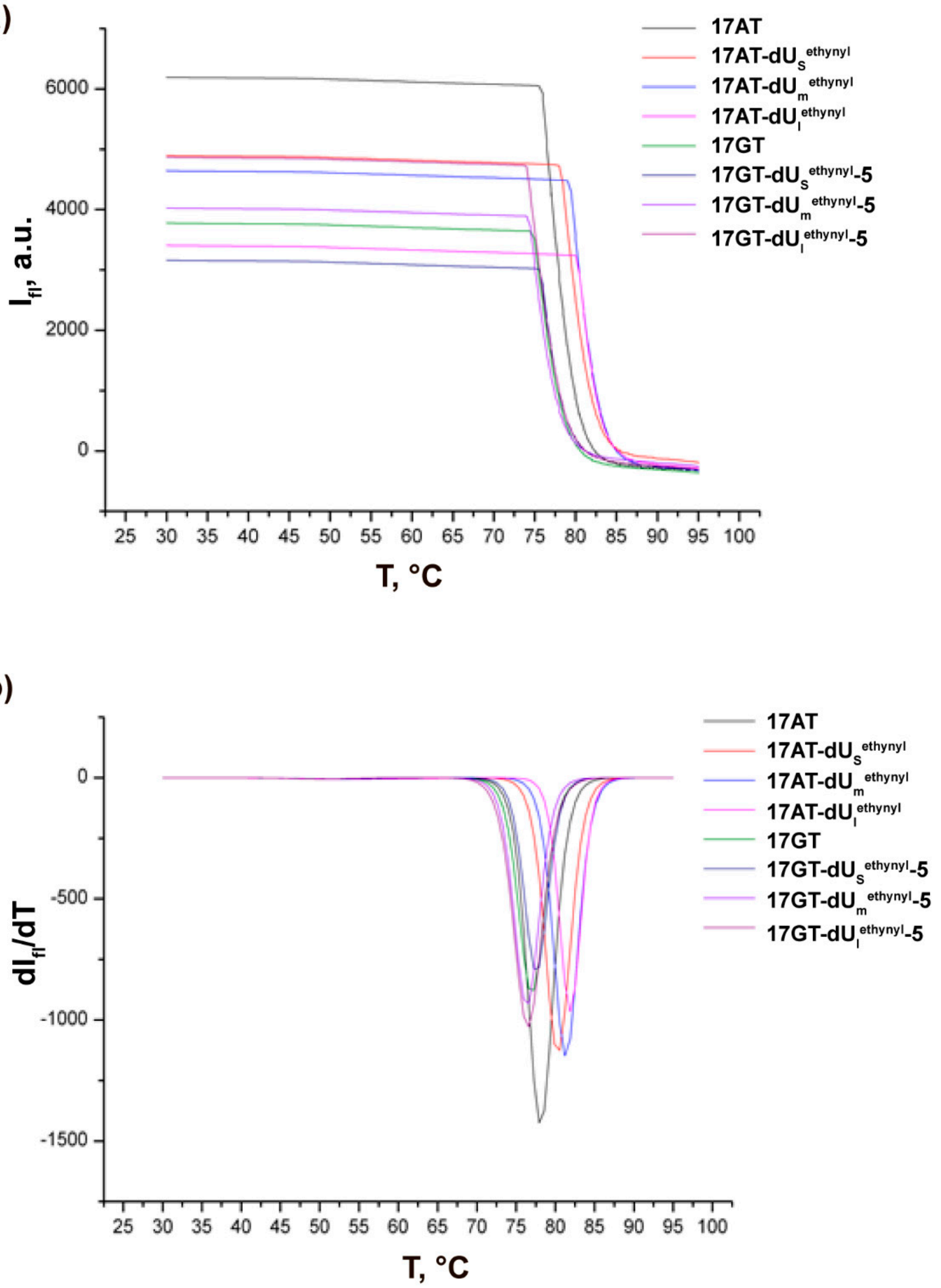

Figure S1. Dependence of fluorescence intensity for DNA duplexes with SYBR Green I on the temperature in integral (**a**) and differential (**b**) forms.

Table S1. Calculated distances (Å) from SG atom of Cys497 and Cys469 in MutS to C5 of thymine at 5th and 8th positions from T of mismatch in the 3ZLJ structure. One letter code is used for amino acids. Subunit number is shown in parentheses.

**Amino acid**	**Distance, Å**	
	in the 5th position	*in the 8th position*
A469 (subunit A)	11	20
A469 (subunit B)	15	13
N497 (subunit A)	16	8
N497 (subunit B)	22	28

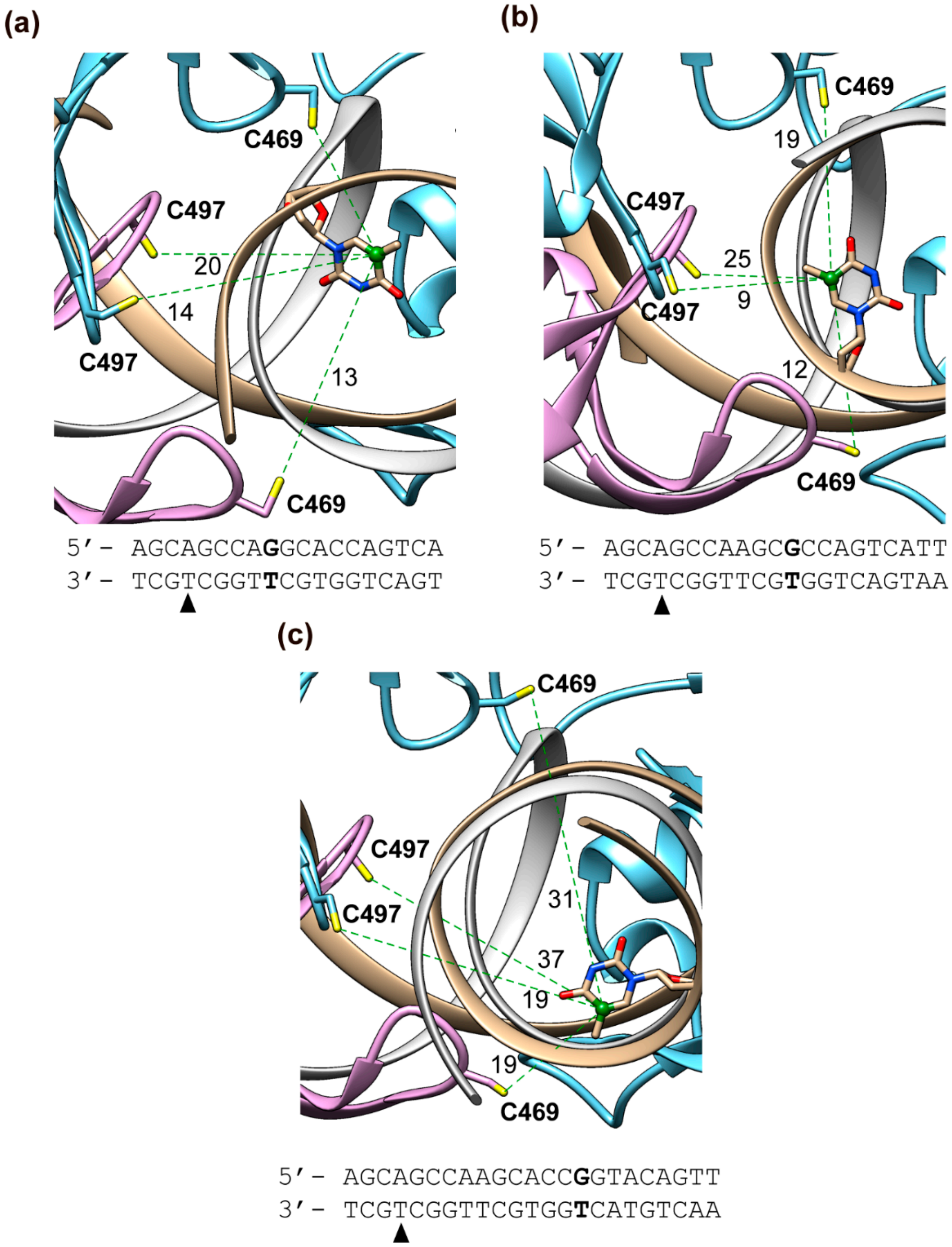

Figure S2. Snapshots of the crystal structure of MutS complex with G/T-containing duplex (PDB code 3ZLJ). The distances from the SG atom of the C469 and C497 to the C5 atom of thymine in 5th, 8th or 11th (panels **a**, **b** and **c** respectively) position from mismatch are indicated (Å). Two subunits of the protein are indicated in blue (subunit A) and pink (subunit B), and DNA in brown. The arrow in the duplexes below indicates the thymidine residue in the duplex, from which the distance to the amino acid residues was measured.
